# Clinical Review, and Hospital Reports

**Published:** 1839-04-01

**Authors:** 


### Clinical Review.

### ST. BARTHOLOMEW'S HOSPITAL.

Observations of Mr. Lawrence on Gonorrhœal Rheumatism and Gonorrhœal Ophthalmia.*

1. *Gonorrhœal Ophthalmia.*—" Gonorrhœal affection of the eye appears in two forms; namely, inflammation of the conjunctiva, with puriform discharge; and inflammation of the external proper tunics, together with the iris.

The former affection appears in various degrees, from a mild and easily manageable disease to the most acute and rapidly destructive inflammation that can affect the eye. The case of Branch exemplifies the active modification, but not its most acute form. It shews that strong astringents, which are useful in the milder cases, are sometimes quite inapplicable in the more dangerous affection, even after considerable depletion. This case would no doubt have ended more quickly, and the patient would have been less weakened, had the plan of incisions through the chemosed conjunctiva, recommended in the valuable paper of Mr. Tyrrell, published in the Medico-Chirurgical Transactions, vol. xxi., been adopted. I have resorted to this plan lately, with excellent effect, in a private case, when the complaint came on in a week after the commencement of gonorrhœa, and seemed to have been caused by some of the patient's urine spirting into his eye. I did not see the case till the third day, when the incisions were immediately practised. A deep ulcer of semicircular figure, formed in the upper part of the cornea, near its edge; and the iris is now adherent to the cornea at that part, but vision is perfect.

In gonorrhœal inflammation of the external tunics and iris, the colour of the latter is altered; there is more or less pain, with imperfection and sometimes temporary loss of sight. These symptoms, however alarming, are not in general attended with lasting mischief to the organ, which may go through several attacks, as in the case of the gentleman above related, without permanent injury to its structure or functions."

So far as we have seen, our experience coincides in the main with that of Mr. Lawrence. But there is one point on which we would offer a remark or two. He observes that strong astringents are sometimes quite inapplicable in the more dangerous forms of gonorrhœal ophthalmia, even after considerable depletion. No doubt this is true, yet it seems to us that this species of faint praise is rather more damning to astringents than they deserve. We have seen extremely acute cases of gonorrhœal ophthalmia benefited remarkably by strong solutions of the nitrate of silver and by the ointment containing it, concurrently with general or local depletion, or after its adoption. In Mr. Lawrence's work on the ophthalmiæ, the value of extreme depletion is certainly over-rated, and that of strong astringents depreciated.

It has appeared to us that the tendency to gonorrhœal ophthalmia, as well as to gonorrhœal rheumatism, prevails in certain families. We are in the habit of attending two brothers, each of whom has had gonorrhœal ophthalmia and rheumatism, separately or conjointly several times. It has also appeared to us that persons prone to rheumatism independently of gonorrhœa, are more prone to rheumatism with gonorrhœa than others are.

---

* Med. Gaz. Jan. 5, 1839.

2. *Gonorrhœal Rheumatism.*—" Although the disease is painful and sometimes obstinate, like ordinary chronic rheumatism, it comes to a conclusion sooner or later, and the patient recovers perfect use of the affected joints. I have met with one exception to this observation, in almost the only case which I have seen in the female. It was that of a robust Irish girl, about 20, who came into St. Bartholomew's with a severe clap and inflammation with swelling of the knee. The joint became and continued highly inflamed, greatly swollen, most acutely painful, with the leg drawn up so as to bend the knee beyond a right angle, in spite of the most active treatment, embracing every measure that could afford any chance of relief. After some months the inflammation and swelling abated under the use of blisters, which were more serviceable than other means; the joint returned to its natural size, but was completely anchylosed in the bent position. As the knee was recovering the elbow became affected: here the disease was less in violence and duration, but the joint remained with considerable limitation of motion.

In its active period gonorrhœal rheumatism requires the same treatment as other acute affections of joints; and it is subsequently benefited by blistering and other modes of counter-irritation. The internal remedies which I have found most useful after the active symptoms have been subdued are the hydriodate of potash, and the compound decoction of sarsaparilla, given in combination. I have usually administered four or five grains of the former in two ounces of the latter three times a day. I lately saw this plan very decidedly advantageous in a case where the feet, knees, and joints of the upper extremities, had been seriously affected, the feet having been swollen and red, and the other joints enlarged and very painful. Mercury had been tried without benefit to the local affections, and with injury to the general health. The hydriodate and sarsaparilla produced an immediate beneficial change; improvement went on steadily, the muriate of morphine being sometimes taken at night; and recovery was effected by continuing the remedies about six weeks, some weakness of the affected joints, with occasional pain, still remaining, although the patient was able to resume his ordinary occupations."

The iodide of potassium with sarsaparilla is undoubtedly a very valuable remedy in gonorrhœal rheumatism which has lapsed into a chronic form. But we have seen it fail to give permanent relief notwithstanding, and Mr. Lawrence's approbation of it is qualified.

We have seen the warm douche and shampooing cure a case which had resisted all internal, and all other external remedies.

---

### Clinical Observations in Surgery. By N. R. Smith, M.D. Professor of Medicine in Transylvania University.*

### I. Extirpation of the Parotid Gland.

On the 21st. August, 1835, Dr. Smith examined, with Dr. Bond, a tumor on the face of Miss Bryan of Baltimore County. It was located between the left ear and the angle of the jaw, in the precise situation of the parotid gland. It presented an abrupt eminence, something in form like a pointing phlegmon—its base not broad. The tumor was hard, occasionally affected with lancinating pains, and tender to the touch. It very much disfigured the patient, and was stated to be increasing in a degree which caused much anxiety.

Both Dr. Smith and Dr. Bond regarded the tumor as originating in a lym-

---

* American Journ. of Med. Sciences, Nov. 1838.

phatic ganglion lying on the parotid, and they concurred in recommending its extirpation.

"In the presence of several of my pupils, August 25, 1836, I commenced the operation by making a vertical incision from the zygoma to the angle of the jaw; and deepening it, laid bare the external aspect of the tumor. On endeavouring to define its lateral limits with the knife, I soon discovered that I had to deal with the entire parotid, and proceeded accordingly. The disease affecting the organ had, in regard to consistence and form, distinguished itself from the surrounding parts. It was much more globular than the healthy gland—had a more distinct envelop of cellular tissue—and had receded in a degree from its confined situation. Penetrating the posterior part of the tumor near its surface, I soon traced out the facial nerve (portio dura) and separated it from the tumor to a considerable extent. I then doubted whether to attempt to disengage the tumor from beneath the nerve, or to divide the latter. Anticipating great embarrassment in the execution of the first plan, and fearing that serious irritation would be necessarily inflicted upon the nerve, I at once divided it. Paralysis of the muscles of the face on that side instantly resulted.

I then cautiously proceeded with the dissection of the tumor. Penetrating between its diseased lobules on every side were occasional bands of cellular tissue. These I divided with great caution, as I expected to find some of them involving the branches of the external carotid, which emanate from the parotid. Thrusting the index of my left hand beneath the tumor, I made them successively turn over its extremity; and carefully feeling for pulsation, I effected their division, sometimes with the knife, but more generally with a very narrow probe-pointed bistoury. When I felt pulsation I endeavoured to effect the laceration of the band with the finger, or the handle of a scalpel. Thus I proceeded till I had insulated the tumor with the exception of a single band attaching the upper and posterior part of the diseased mass to the deep temporal region. Occasionally there had sprung a small artery, but not furnishing sufficient blood to embarrass the operation. I now divided the last band which attached the tumor, and a single artery sprung with considerable impetuosity. This I secured without difficulty with the tenaculum. Had I felt any considerable pulsation in it I should have included the whole band in a ligature before effecting its separation.

The tumor being now removed, I explored the cavity from which it had been taken. This extended quite to the styloid process; and the muscles arising from that point were seen with perfect distinctness. Not a vestige of any thing presenting the appearance of the parotid gland could be seen in the space usually occupied by it. Probably, however, that small process of the gland which extends forward on the cheek, termed socia parotidis, was left; but in the incision along the anterior border of the tumor I did not distinguish it or the duct of Steno. The tumor is in my possession, and is of the size of a very large hickory nut."

The wound healed satisfactorily, though the paralysis of the face was permanent. In the course of some months the latter "had decidedly diminished."

Dr. Smith thinks that this case tends to reconcile the discrepancies between anatomy and practice in reference to the removal of the parotid gland. Anatomy seems to say that it is impossible—practice that it has been done.

The feasibility of the operation is, in Dr. Smith's opinion, to be explained by the facts furnished in the above instance. The tumor in its growth had assumed a harder consistence than natural, without having imparted disease to the surrounding parts. It was therefore better defined than the healthy organ. It had also become spheroidal; and from its size and hardness had necessarily receded from its confined situation. Its extirpation was therefore undoubtedly far easier than would be that of a healthy gland; and, because of the obliteration of the vessels from causes named above, attended with far less hæmorrhage.

### 2. Successful Amputation for Traumatic Gangrene.

Aug. 31, 1837.—The patient was a youth 17 years of age, of good constitution, but probably at the time of the injury, somewhat under the influence of malaria, as he came from a sickly district on the eastern shore of Maryland. Being on board a bay craft he suffered a fracture of the leg near the knee, by the fall of a bag of merchandize which was being removed from the vessel.

Dr. Smith saw him on the fifth day from that of the injury. The limb was then reposing on pillows, in the semiflexed position, not having been placed in splints. The whole foot and leg, to within three inches of the knee, were in a state of complete mortification, the parts being tumid, crepitous when pressed, covered with dark vesications, cold and completely insensible. A belt of gangrenous inflammation existed below the knee; but nothing like a line of demarcation existed between the dead and living parts. There was considerable tumefaction at the place of fracture; but the action above the knee was neither excessive nor unhealthy. His pulse was firm and good, (about 100), the nervous system but little disturbed, and the stomach performing its offices as well as in ordinary cases of fracture.

The consultants came to the conclusion that the mortification must have resulted from some local cause; probably some lesion inflicted upon the great vessels and nerve, and they determined to amputate above the fracture. In less than two hours, Dr. S. performed the operation, and the boy recovered rapidly.

The fracture was within about two inches and a quarter of the extremity of the bone, and perfectly transverse. The upper fragment was thrust into the ham, and lodged directly behind the lower fragment, which it overlapped for about three-fourths of an inch. In the midst of effused blood and serum were found the femoral artery and vien thrust backward, and tensely drawn across the sharp posterior margin of the superior fragment, in such a manner that it was perfectly obvious that the circulation in both vessels must have been completely interrupted.

Of course, in such a case, the chances of success from an operation are much greater than in an ordinary instance of traumatic gangrene.

---

### MASSACHUSETTS GENERAL HOSPITAL.

### Cases of Pneumo-Thorax, and Experiments to determine the Causes of "Metallic Tinkling." By J. Bigelow, M.D.*

Dr. Bigelow relates three cases of pneumo-thorax, and has instituted six experiments, for the purpose of determining the vexata quæstio—the cause of the "metallic tinkling." It is not necessary to enter on the cases, but we shall present our readers with Dr. Bigelow's account of the experiments, and his conclusions.

*Experiment* 1.—Previously to the autopsies of the patients who were the subjects of Cases 1 and 2, a glass cylinder, open at both ends, was pressed into close contact with the chest, so as to hold water. Some ounces of that fluid were poured in, and a perforation was made through it, into the cavity of the chest on the distended side. Immediately a large volume of air escaped from the chest,

---

* American Journ. of Med. Sciences, Nov. 1838.

bubbling upwards through the water. In the third case, no cylinder being at hand, a superficial cavity was made out of the dissected integuments of the chest, and filled with water. Through this water a perforation of the chest was made on the left anterior surface. The air rushed out, producing strong ebullition, as in the former cases. The experiment was then repeated on the right side, and the perforation made through water as before. No air in this instance escaped, but the water was immediately sucked into the chest by the atmospheric pressure.

*Experiment* 2.—Artificial respiration was produced in the body of the subject of Case 2, by inflating the lungs through the trachea, and expelling the air by pressure on the abdomen. At each inflation, a most distinct, clear and abundant metallic tinkling was produced, accompanied with more or less amphoric sound, and could be sustained ad libitum by repeating the inflation.

*Experiment* 3.—Through an aperture in the anterior part of the chest in the subject of Case 2, a catheter was introduced and air blown through it into the cavity of the left pleura. While the end of the catheter was above the level of the fluid, a strong amphoric buzzing was communicated to the ear of an observer in contact with the chest. But when the end of the instrument was pushed below the surface of the liquid, and the latter made to bubble by continuing the inflation, an exquisite metallic tinkling was heard at the explosion of each bubble, resembling, as it had done in life, the sound of a little bell or musical wire. In the subject of Case 3, this experiment was repeated, and varied by pouring into the chest different quantities of water. When a few ounces only were present, metallic tinkling was uniformly produced, but when two quarts or more were introduced, a bubbling only was heard, without metallic resonance. Similar results were also obtained by pouring a small stream, or letting fall drops of water from above, upon the liquid in the chest.

*Experiment* 4.—Succussion and percussion were both found to produce the same metallic sounds in the dead body as during life in Case 2. Metallic sounds elicited by percussion somewhat resemble those occasionally yielded by the heart, and, as has been observed by Bouillaud, these may be imitated by percussing the back of the hand pressed closely upon the ear, or by closing both ears with the palms of the hands, and walking on a carpet in a still room.

*Experiment* 5.—In the body of a person recently dead from accident, having no pneumothorax, a repetition was made of several of the foregoing trials. Air and water were forced into the chest, the former so as to distend the cavity and render percussion quite resonant. Ebullition of the fluid was then produced by blowing through a tube inserted between the ribs and pushed below the surface. The only result was a bubbling noise, having not the slightest metallic character. It will be observed that this was nearly a repetition of Magendie's experiment, and it probably failed to produce metallic sound for the same reason as in that case, viz. that the patient was not pneumothoracic.

*Experiment* 6.—A bladder, and afterwards a stomach, each containing a few ounces of water, were inflated until thoroughly distended. Whenever the inflating tube was pushed below the surface of the liquid, and the inflation continued so as to produce bubbles, a sharp tinkling was heard upon the explosion of every bubble, by the ear applied as in auscultating to the outside of the bladder. In this experiment the sound becomes more exquisitely metallic, in proportion as the tension of the bladder is increased by farther inflation. Succussion of the bladder produces a similar effect. It is necessary that a recent bladder should be used, the texture and elasticity of which are not altered by drying. When the orifice of the tube is above the surface of the water, also when no water is present in the bladder, an intense amphoric sound is produced during inflation; and if saliva or other liquid, in small quantities, is blown through the inflating tube, a more feeble, or *sub-metallic* tinkling is produced.

From these experiments, and from the cases to which they are pendents, Dr. Bigelow concludes that the following agencies are concerned in producing metallic sounds of the chest.

1. "There must be a cavity, the walls of which are preternaturally susceptible of vibration. This takes place when the pleura is pathologically distended, so as to overcome the obtuse or muffling effect of the contiguous soft organs, such as the lung, diaphragm and intercostal muscles. Some time is probably necessary to prepaie the parts for this pathological resonance, since it fails to appear *post-mortem* in healthy chests submitted to experiment. It should be added that when metallic sounds appear in simple phthisis, there are cavities of the lungs, the walls of which are in a state of tubercular induration.

2. The immediate or exciting cause of metallic tinkling, is a forcible or sudden disturbance of the liquid in a vibrating cavity like that described. The explosion of bubbles of air from beneath the surface of the liquid, appears to be the most common cause of such a disturbance; but it may also take place when a part of the liquid is thrown upward in the act of coughing and falls back upon the remainder. The same occurs in succussion of the chest.

3. The vibrations which yield metallic tinkling are transmitted from the liquid to the solid parietes, and thence directly to the ear, without any necessary agency of an echo, or a reverberation of air in the cavity. This is shown particularly by the experiment of the bowl.

4. A minor, or *submetallic* tinkling, having no musical resonance, may be produced by slight impulses given to the air in the cavity, such as the breaking of bubbles of mucus at orifices above the surface of the liquid.

5. Amphoric resonance is produced by reverberations of the air in a vibrating cavity, without sonific impulse of the liquid. The same is true of metallic modifications of the voice, and of the cough when there is no tinkling. Metallic percussion seems also to depend upon the vibrations of air independently of liquid, and may be produced in some other cases when we strike upon a tense cavity in which a certain quantity of air is confined."

---

### CLINICAL REPORTS FROM THE PENNSYLVANIA HOSPITAL.*

### I.—DISLOCATION OF THE HUMERUS, REDUCED AT THE END OF TWENTY-ONE DAYS.

Eliz. B. a servant, aged 22, had laboured under an unreduced dislocation into the axilla for twenty-one days, when reduction was effected in the hospital, by Dr. Norris. The following, not unusual, method was pursued.

The patient having taken a grain and a half of tartar-emetic, in divided doses, Dr. Norris proceeded at once to the reduction. A folded sheet was firmly applied by wet rollers to the arm, just above the elbow, to effect extension with the use of the pullies; another sheet was passed around the chest, and secured to a staple, for counter-extension; and in order firmly to fix the scapula, a third band was applied over the acromion process, and given to assistants, who were directed to apply their force in a line obliquely downwards towards the opposite

---

* Medical Examiner, Philadelphia.

side. The apparatus being adjusted, a vein was opened, and extension kept up by two assistants, for the space of about fifteen minutes, at the end of which time all extension being suddenly removed, the bone was found to be reduced.

### II.—Dislocation of the Head of the Radius Backwards, reduced.

"Mr. John B. W——, clerk, aged 23 years, whilst riding in the city, on the afternoon of the 12th of May, accidentally fell from his horse and dislocated the left radius backwards. He stated 'that he fell on his left hand whilst it was turned inwards, and immediately felt a sharp pain in the elbow, which induced him to think he had broken his arm.' He came at once to the hospital; the arm was semiflexed; he was unable to supinate the hand, or to bend the elbow or straighten it, without intense pain. The head of the radius was distinctly felt behind the external condyle of the humerus, resting against the olecranon process. On partially pronating and supinating the hand, the head revolved distinctly, and owing to the little swelling, and the slight development of his muscles, the button shape of the radius was distinctly seen revolving against the olecranon. The patient, to use his own expression, had always been very 'loose-jointed,' but had never before dislocated any of his bones. After a close examination of the case, he was placed on a bed; an assistant was directed to seize the humerus near the condyles, place his thumbs against the head of the bone, and force it downwards and forwards. At the same time, I made extension at the wrist, suddenly straightened the arm, and supinated the hand, when the bone readily slipped into its place. The arm was then placed in a carved angular splint, loosely bandaged, and cold lotions applied to it. The next day, the patient could bend the arm, and perform the usual motions of the hand, but, of course, was not allowed to do so. Little inflammation followed, and on the 14th of May, still wearing the splint, he returned to Mullaga Hill, N. J. where he resided."

Our readers are aware that this accident is exceedingly uncommon. The reduction appears to have been effected with precision and dexterity.

### III.—Three Cases of Compound Fracture of the Cranium.

Our surgical readers are aware that some difference of opinion exists on the treatment of "compound fracture with depression of the cranium." While Sir A. Cooper, Sir B. Brodie, and some other surgeons, recommend trephining as a rule in this accident; others, for example, Mr. S. Cooper, do not acquiesce in the general principle on which that advice is founded. We notice the following cases in order to add them to the recorded facts, which form the best data for generalization.

*Case* 1. Joseph W——, æt. 10 years, was struck on the head by a large block of wood, which fell from a three story dwelling, on the 10th August, 1837. On his entrance, three hours afterwards, he had a wound of three and a half inches in the scalp, bone bare of periosteum for two inches, fractured the length of the cut, and depressed about an eighth of an inch. Patient insensible; pulse strong, corded and slow; pupils dilated, extremities cold. He was trephined immediately by Dr. Norris. On raising the bone consciousness instantly returned. The middle artery of the dura mater was cut by the trephine, but the hæmorrhage was checked by a coagulum, which soon formed. The scalp was drawn partially together by adhesive strips, and the wound poulticed—ordered perfect rest in a dark room, and lowest diet. Evening.—Pulse increased, skin hot and dry—lies quiet, and complains little; ordered V. S. ad f℥vi, and mist. sal. f℥vi. ant. tart. gr. ss., a table-spoonful every two hours.

On the 31st, there was considerable fever. *V. S. ad* ℥x.—*Hyd. Sub. gr.* ij. 4*tis horis.*

This removed the febrile excitement. The wound suppurated, granulated, *threatened* fungus cerebri, (compresses of lint soaked in lime-water prevented its occurrence), several pieces of bone came away, and on Nov. 5, the patient was discharged cured, the wound having firmly healed and the intellects being unimpaired.

*Case* 2. This case was too severe, and the issue too rapidly fatal, to admit of conclusions respecting the value of modes of treatment. The injury happened from a man falling head-foremost against a large circular saw, which was revolving 240 times in the minute. A fur cap on his head was torn to tatters, the scalp terribly lacerated, and turned back, leaving the skull bare for a space of four inches long by three wide; the skull was fractured from the lower part of the os frontis to the vertex, in a line a little oblique to the sagittal suture. The saw had penetrated the dura mater, a process of which was hanging out, and entered deeply into the substance of the brain. The patient was sensible, and could sit up in bed. But he became delirious in the night, comatose next day, and died within forty-eight hours after the injury.

Among other lesions, there were—the arachnoid much inflamed and thickened; several drachms of pure pus found on the brain and membranes; vessels injected; brain very soft around the wound; wound in the anterior lobe of left hemisphere of cerebrum, two and a half inches long, and near two inches deep; brain covered with pus, and much softened on that side.

The early occurrence of suppuration is sufficiently striking to induce us to give this brief account of the case.

*Case* 3, was one of pistol-ball lodged in the brain, which proved fatal on the third day, with such symptoms as might naturally be expected to occur. It is not, we think, necessary to detail the case.

The only instance of the three which bears upon the question of trephining, in cases of compound fracture of the cranium with depression, is the first. In that it will be observed that suppuration occurred, and it is fair to suppose that had the bone not been raised and removed by the trephine, the collection of pus might have produced unpleasant consequences.

### IV.—MORTALITY AFTER AMPUTATIONS, IN THE PENNSYLVANIA HOSPITAL.

Dr. Norris, one of the surgeons of the hospital, has made a report on this subject. He says:—

" Contrary to the opinion generally prevalent in this country, amputation, even under favourable circumstances, is frequently followed by fatal results in civil hospitals. In the practice of the Hôtel Dieu, of Paris, it is said that not more than half of the cases prove successful; and I have the authority of M. Hache, a former interne of the hospital of St. Louis, of the same city, for stating, that out of twenty successive amputations made in the year 1833, in that institution, twelve died.

After a tabular view of the operations performed in the Pennsylvania Hospital, for the last seven years, Dr. N. observes:

" Of the above 56 amputations on 55 patients, 24 were primary, of which 14 were cured, and 10 died; 4 of the deaths occurring within the 24 hours immediately following it; 12 were secondary, of which 5 were cured and 7 died; 20* were for the cure of chronic affections, of which 15 were cured, and 4 died;

* One of the patients here included suffered double amputation.

23 of the amputations were of the upper extremity, of which 18 were cured and 5 died; 33 were of the lower extremity, of which 17 were cured, and 16 died; 6 were amputations at the joints, of which 4 were cured, and 2 died.

Of the 55 patients operated on,

9 were under 20 years of age, of whom 8 were cured and 1 died.
21 between 20 and 30—15 were cured, and 6 died.
16 between 30 and 40, 9 " 7 "
9 between 40 and 50, 3 " 6 "

From this resumé of seven years' practice at the Pennsylvania Hospital, it appears,

1st. That amputation is to be regarded as an operation attended with much danger to the life of the individual.

2d. That the chances of success after it, are much greater in persons who have been for some time suffering from chronic diseases, than in those who have it done whilst enjoying robust health.

3d. That amputation of the lower extremity is much more fatal than that of the superior member, and

4th. That the danger increases with the age of the individual operated on."

We may here introduce some statistical observations by Mr. Benjamin Phillips, read before the Medico-Chirurgical Society, in November, 1837, and published in the Medical Gazette for June, 1838. To these observations our contemporary, the Examiner, alludes, and we quote from his quotations.

"I have now shown that the mortality succeeding to amputation is very great—23 per cent. I shall therefore proceed to analyse the gross number, and exhibit the proportion furnished by the different countries implicated in the inquiry. They are as follow:

| | Cases. | Deaths. | | Per Cent. |
|---|---|---|---|---|
| France | 203 | 47 | or | $23\frac{31}{203}$ |
| Germany | 109 | 26 | | $23\frac{93}{109}$ |
| America | 95 | 24 | | $25\frac{5}{19}$ |
| Great Britain | 233 | 53 | | $22\frac{174}{233}$ |
| | 640 | 150 | | $23\frac{7}{16}$ |

Here is an average number of deaths, amounting to, as near as may be, 23½ per cent. If the several countries be taken separately, we find that France is a fraction below this average; that Germany differs only to the amount of a fraction from France; that America only exceeds the average by a little more than 2 per cent.; and that Great Britain is a fraction below the average."

The main object of Mr. Phillips is to shew that the mortality after amputation is much greater than is usually believed.

Mr. Phillips justly observes, that if compared with the much dreaded operation of lithotomy, amputation is much more fatal.

Mr. Phillips thinks it proved that there is a large class of cases, in which union by the first intention is attended with more danger, than when suppuration is permitted. This class comprises diseased articulations.

"Of these cases, immediate union was attempted in 117; consecutive union in 96.

Of the 117 cases, 88 only succeeded; the deaths amounted to 29.

Of the 96 cases, in which the treatment was by consecutive union, 76 succeeded; the deaths were 20.

Of these cases, Great Britain furnished 86; the other countries included in the observations, 127.

Of the 86 cases, immediate union was attempted in 60; consecutive in 26, and with the following result: of the 60 cases there were 15 deaths; of the 26 there were 5 deaths.

Of the 127 cases, immediate union was attempted in 57 cases; consecutive in 70.

Of the 57 cases, 14 were unsuccessful, the patients died, and 43 succeeded. Of the 70 cases where consecutive union was employed, there were 15 deaths. The results, therefore, attendant upon the practice of immediate union, are a mortality amounting to 25 per cent.; upon consecutive union, of nearly 21 per cent.

And there is a singular uniformity attendant upon the results of these two modes of practice, as shown by the returns furnished by our own and the other countries; and all are strongly confirmatory of the prudence of avoiding immediate union in this large and well-defined class of diseases."

---

STATISTICAL REPORT OF THE RICHMOND LUNATIC ASYLUM. By JOHN MOLLAN, M.D. Physician Extraordinary to the Asylum. Octavo, stitched. Dublin, 1838.

The attention of the profession scarcely requires to be directed to insanity. Yet there are many points connected with it, which are understood imperfectly, and so much hinges on a sound acquaintance with its phenomena and management, that medical men cannot study them too closely.

The present report is far from uninteresting. Our brethren in Ireland are beginning to exhibit a degree of zeal and activity which bode the best results to science. The officers of charitable institutions are particularly zealous.

It appears that the Richmond Lunatic Asylum was completed in the year 1814. It was planned not simply for the safe-keeping of the insane, but with a view to their rational treatment and cure.

For many years after its opening, patients were admitted into the Richmond Asylum from all parts of Ireland; but since the year 1830, when the various District Asylums were completed, it has been appropriated to a certain district, which embraces the city and county of Dublin, the counties of Meath, Louth, and Wicklow, and the town of Drogheda. A residence of twelve months within the district is necessary to entitle a patient to be received, and none are admitted without a medical certificate of insanity, and an affidavit on the part of the nearest relative, that the individual is a pauper, by which is understood, not being possessed of sufficient means to pay for maintenance in a private asylum. A few persons have been occasionally admitted, who contributed small sums to the funds of the institution; but the number of such cases at one time in the house, has rarely amounted to eight; and it is right to state, that they are treated in every respect like the general class of patients.

Cases of a manifestly incurable character are not admissible, but this rule can never be strictly enforced. Persons subject to epilepsy are excluded by the same regulation, but they sometimes get admission from the existence of the disease being concealed.

The plan of the building admits the formation of five classes for each sex, and a separate airing-ground is appropriated to each division. An important addition has lately been made of a large piece of ground, and including the gardens originally attached, there are now nearly twenty English acres belonging to the asylum.

The house was planned originally for the reception of 236 patients, but by alterations subsequently made, 288 can now be admitted; and yet, owing to the accumulation of incurable cases during a series of years, the accommodation is found to be inadequate to meet the wants of the district, and the Asylum labours under the disadvantage of not being at all times able to receive patients immediately on their being attacked, a circumstance which has an important effect on the result of treatment. But this defect is likely to be soon removed, Government having sanctioned the addition to the building of accommodation for 100 patients.

Dr. M.'s Report embraces a period of five years, commencing January, 1833, and ending December, 1837.

The population of the district, according to the census of 1831, amounted to 803,396, and each county being charged with the expense of the patients received from it, Dr. M. is enabled to compare the numbers with the population of each division respectively.

| Admitted from | Population. | Number of Patients. | |
|---|---|---|---|
| | | Males. | Females. |
| City of Dublin (Within the ancient boundaries.) | 204,155 | 167 | 128 |
| County of Dublin | 176,012 | 68 | 80 |
| County Meath | 176,826 | 40 | 25 |
| County Wicklow | 121,557 | 25 | 23 |
| County Louth | 107,481 | 27 | 19 |
| Town of Drogheda | 17,365 | 4 | 2 |
| | | Total, 331 | 277 |

The large number of patients admitted from the city and county of Dublin, as compared with the other divisions, shews the much greater prevalence of the causes of insanity in cities and thickly populated neighbourhoods, than in the rural parts of the district. The town of Drogheda is rather an exception to this rule.

The medical certificate required with each patient contains a query as to the probable cause of the disease, where such could be assigned. From this source Dr. M. has arranged a table of causes.

| | M. | F. |
|---|---|---|
| Intemperance and abuse of ardent spirits | 74 | 12 |
| Pecuniary losses and reverses of fortune | 35 | 20 |
| External injuries from falls, wounds, &c. | 18 | 4 |
| Destitution and want of employment | 13 | 10 |
| Domestic disagreements and bad treatment by relatives | 9 | 10 |
| Love and disappointed affections | 5 | 18 |
| Jealousy | 10 | 10 |
| Seduction | 0 | 1 |
| Religion | 10 | 18 |
| Pride | 1 | 1 |
| Excessive fondness for music, | 1 | 0 |
| Grief from death of relatives, | 11 | 18 |
| After fever | 10 | 8 |
| cholera | 1 | 1 |
| influenza | 1 | 0 |
| scarlatina | 0 | 1 |
| erysipelas | 0 | 1 |
| Apoplexy | 2 | 0 |
| Dread of cholera | 0 | 1 |
| Abuse of mercury | 10 | 2 |
| Fright | 4 | 26 |
| Hereditary predisposition | 8 | 5 |
| Close confinement and study, | 7 | 0 |
| Political excitement | 1 | 0 |
| Puerperal state | 0 | 22 |
| Violation of the person | 0 | 3 |
| Change of life consequent on marriage | 0 | 1 |

"This table shews a greater proportion of physical causes amongst men, and of moral causes amongst women, not exclusive of those peculiar to the latter sex. Besides the large number of cases attributed to intemperance, I have no doubt that the same cause operated remotely in many others, by leading to those circumstances from which the disease was supposed more immediately to proceed. Of the cases denominated religious, I believe but a very small number could fairly be attributed to religion as a cause; religious enthusiasm and religious despondency, though often mistaken for the causes, are much more frequently the consequences of insanity. The puerperal cases occurred either during pregnancy or very soon after delivery; in some few of them, other causes may have been combined with the puerperal state, such as fright and family disagreements. One woman who was attacked immediately after the birth of her first child, had been insane before marriage. In addition to those cases stated as hereditary, wherein this was the only cause assigned, nineteen others were noted, one or other of whose parents had been insane; and in forty-four more, insanity had shewn itself in collateral blood relations. Amongst the cases for which no satisfactory cause was assigned in their certificates were two young females, aged sixteen years, in whom the catamenia first appeared after being admitted into the asylum, and they speedily recovered on the establishment of this discharge. One was readmitted at the end of eight months, and the other after an interval of more than three years, both being affected with suspension of the catamenia. Suitable means being employed for the restoration of this evacuation, every symptom of insanity soon disappeared. In many other cases, irregularities of the menstrual function existed; in some, perhaps, the consequences, in others, very probably, the cause of their insanity; but in none was the connexion of the two affections so strikingly exhibited as in the two first mentioned."

Dr. Mollan earnestly and properly presses on those gentlemen who sign certificates of insanity, the necessity for accuracy in filling up answers to the queries they contain. Those answers are to form the basis for generalizations. How requisite their truth must be.

The occupation or condition in life of each patient is shewn in the table following.

| MALES. | | FEMALES. | |
|---|---|---|---|
| Clergymen | 3 | Governesses | |
| Apothecaries | 2 | Schoolmistresses | |
| Attorneys | 1 | Shopkeepers and small traders | |
| Traders and shopkeepers | 23 | Dressmakers and other female trades | |
| Schoolmasters | 5 | Domestic servants | |
| Mercantile and law clerks | 27 | Washerwomen | |
| Artisans and tradesmen | 132 | Wives or daughters of tradesmen, | |
| Servants | 32 | Do. of professional men | |
| Farmers | 11 | Do. of farmers | |
| Soldiers and pensioners | 9 | Do. of labourers | |
| Sailors | 9 | Do. of clerks or stewards | |
| Police and revenue officers | 6 | Mendicant | |
| Labourers | 60 | Midwife | |
| Mendicant | 1 | Prostitutes | |
| Unknown | 10 | Unknown | |

The class of labourers, the lowest, most numerous, and most likely to furnish tenants to a pauper asylum, only gives a fourth of the cases. Insanity prevails more in the middle and upper, than in the lower classes.

"In my inquiries respecting the condition in life of the females, I was surprized to find so small a number of prostitutes, as the contrary might be expected, on considering the excesses to which they are addicted, and the moral and physical evils to which they are exposed. Only three have been admitted within the last five years; and I learn that the average number in former periods was not greater. It is right to anticipate an objection that may be made, by stating, that women of this class, when proper subjects for the asylum, find as ready admission as any others. I should not have alluded to this circumstance, but that it is at variance with reports of lunatic asylums elsewhere, particularly in Paris. It has been stated by Esquirol, that at the Salpêtrière, the great hospital for insane females, one-twentieth of the whole number had been prostitutes. And Parent Duchatelet, in his extraordinary work on prostitution in the city of Paris, states that 105 cases of insanity had occurred amongst prostitutes in the course of five years. Why women of this description in Dublin should be more exempt, I can assign no satisfactory reason. The peculiar manners and habits of the same class in the French capital, as described by Parent Duchatelet, will probably account, in some degree, for their greater liability."

Of the men, 126 were married, twelve were widowers, and 134 were unmarried. Ninety-eight of the females were married, twenty were widows, and 115 were unmarried. The state of the remainder was not ascertained.

"An account being kept of the religion of the patients; of the 608 under consideration, 146 were Protestants, and 462 Roman Catholics, being in the proportion of one of the former in every $4\frac{1}{6}$; which is greater than that of the respective numbers in the population at large, wherein, according to the census, Protestants constitute one in every $5\frac{1}{4}$. But as all classes are included in this calculation, a fairer comparison would be of the patients in the asylum with the admissions into a general hospital indiscriminately open to the lower classes. I have, accordingly, procured returns from two of the hospitals connected with the House of Industry, of the patients admitted in one year, and I find the Protestants to have been one in every seven. I am, therefore, warranted in saying, that relatively to the respective numbers in the population, there are more insane Protestants than Catholics. This difference I conceive to have very little connexion with religious belief, but is mainly, if not entirely attributable to the fact, that the great disparity in the numbers of Protestants and Catholics in this country exists in the lowest classes of society, and that in ascending the scale, the numbers approximate; and as it has already been stated, insanity is least prevalent in the lowest classes. It is not meant by these observations altogether to exclude religion as a cause of insanity; and perhaps cases of a purely religious origin may be more common amongst Protestants, than Catholics; they are, however, only of rare occurrence."

TABLE OF AGES.

| | Under 20 years. | 20 to 30. | 30 to 40. | 40 to 50. | 50 to 60. | 60 to 70. | Above 70. |
|---|---|---|---|---|---|---|---|
| Males.... | 17 | 85 | 106 | 75 | 29 | 16 | 3 |
| Females.. | 20 | 85 | 86 | 42 | 28 | 10 | 6 |
| Total .... | 37 | 170 | 192 | 117 | 57 | 26 | 9 |

487 were admitted in the first attack, seventy-eight in the second, twenty-four in the third, fourteen in the fourth, four in the fifth, and one in the sixth attack of insanity.

The duration of the disease before admission is shewn in the next table.

| | ADMITTED IN THE MONTH. | | | | | | | | | | IN THE YEAR. | | | | |
|---|---|---|---|---|---|---|---|---|---|---|---|---|---|---|---|
| | 1st. | 2nd | 3rd | 4th | 5th | 6th | 7th | 8th | 9th | 10th to 12th. | 2nd | 3rd | 4th | 5th | 6th |
| Males.. | 78 | 69 | 33 | 30 | 20 | 10 | 17 | 5 | 7 | 9 | 17 | 15 | 12 | 3 | 6 |
| Females | 50 | 47 | 32 | 28 | 12 | 7 | 13 | 5 | 4 | 12 | 24 | 17 | 9 | 6 | 11 |
| Total.. | 128 | 116 | 65 | 58 | 32 | 17 | 30 | 10 | 11 | 21 | 41 | 32 | 21 | 9 | 17 |

It has sometimes occurred that a delay in the admission arose from the want of sufficient accommodation in the asylum; much more frequently, however, from neglect of timely application. The friends of patients often dislike their being placed in an asylum so long as they are manageable at home, and in consequence, that period of the disease is too frequently allowed to pass over when proper medical treatment is most likely to be efficacious.

The results of treatment are next adverted to.

| | DISMISSED. | | | | |
|---|---|---|---|---|---|
| | Recovered. | Relieved. | Unrelieved. | Died. | Remaining. |
| Males - - | 150 | 29 | 10 | 57 | 85 |
| Females - | 116 | 44 | 3 | 31 | 83 |
| Total - - | 266 | 73 | 13 | 88 | 168 |

From the recoveries, forty-two must be deducted on account of re-admissions in consequence of relapses; thus reducing the number of permanent recoveries to 224, so far as the event is known. But on the other hand, credit is to be taken for at least one-half of those discharged as relieved, who are known ultimately to have recovered; the removal of a patient from the asylum who is tranquil and easily managed, having often a salutary effect. Again, of those remaining, a very considerable number are likely to recover, which circumstance must be considered in forming any calculation of the curability of the disease.

Dr. Mollan adverts to the pathology of insanity, but so cursorily that we do not think it necessary to notice this part of his report. We may simply observe, that many more males died from head-affections than females, a circumstance corroborative of the greater prevalence of the physical causes of insanity in the former.

The tendency of insanity to shorten life is a subject of importance, were it only in reference to life-assurance. Dr. Mollan subjoins the ages at which death occurred, as a contribution to the stock of data.

DIED.

| Between 20 and 25 years. | 25 and 30 | 30 and 35 | 35 and 40 | 40 and 45 | 45 and 50 | 50 and 55 | 55 and 60 | 60 and 65 | 65 and 70 | 70 and 75 | 85 |
|---|---|---|---|---|---|---|---|---|---|---|---|
| 3 | 9 | 11 | 14 | 10 | 9 | 7 | 7 | 9 | 3 | 5 | 1 |

In our last notice of the work of Esquirol, we gave at full length the results of his observations on the value of laborious employment in the treatment of insanity. The following observations so strongly confirm the opinions and statements of that distinguished physician, that we give them at full length. The subject is one of the first importance.

"The advantages of laborious employment in the treatment of insanity have long been known, but its systematic adoption in public asylums is of recent date. I have already mentioned the extent of ground attached to the asylum; the part of this lately acquired, is now being converted into a market garden for the growth of vegetables for sale. An average number of sixty men are constantly employed in the cultivation of the grounds; and although entrusted with the use of spades, shovels, and other implements, no serious accidents have ever occurred. They are of course at all times under proper superintendance. About fifteen men find employment in various trades, as weavers, tailors, shoemakers, and carpenters. A few are occasionally engaged in breaking stones, and making mats, others in the performance of various domestic offices in the house. The female occupations consist of spinning, knitting, and the various branches of needle-work, and they assist in washing, and in all the offices of house-maids. All the clothing for both males and females is made up by the patients, with the exception of hats and shoes. During the last year, 2088 yards of linen and calico were woven in the establishment, the yarn for the linen having been spun by the females, and 524 pairs of stockings were made by them. Small rewards are generally given for the articles manufactured, which here as elsewhere operate powerfully as a stimulus to exertion. Of the advantages of this system of employment, I can speak in very decided terms; persons who, when unemployed, are noisy, violent in their demeanor, quarrelsome, and discontented, very generally become, under the influence of suitable occupation, tranquil, orderly, and easily managed; and to its salutary effects I mainly attribute the recovery of numerous cases, some of which were at first most unpromising. Difficulties will no doubt occur in its individual application, requiring perseverance and address, and of course, it is not suitable to every case, nor to every stage of the disease; but I can confidently say, that at least eight out of every ten lunatics will be found in a fit state for some useful pursuit; and it is often a subject of regret that our means of employment are inadequate. The advantages, in an economical point of view, of employing the patients in a pauper establishment, are so obvious as to require no comment.

I shall give a brief sketch of a few cases in illustration of these observations. A farmer about twenty-seven years of age was admitted into the asylum, after having been nearly four years in a state of dementia, in consequence, as was reported, of the improper use of mercury. He was listless and taciturn, and would only reply to questions about himself, by telling his name and place of abode; his general health was unimpaired, and his appetite was good, but before commencing his meals he always required the assurance of some of the attendants that the food was good, and proper for him to eat. At first, he refused to engage in any occupation, but being otherwise amenable, and shewing no vicious propensity, a wheel-barrow was placed before him, and a man at either side took him each by a hand, and fixing them on the handles of the barrow, he was gently urged to the use of it; finding this plan persevered in, he soon consented to work by himself; and afterwards taking to the use of the spade he proved himself an excellent workman. In a short time an improvement was apparent in his mental state, he became gradually more intelligent and communicative; his recovery proceeded without interruption, and he was discharged quite well after a residence of eleven months in the asylum.—A tailor, aged thirty-six years, was admitted in a state of melancholy of six months' duration; he was remarkably timid and reserved, and when questioned about himself he seemed disposed to give the required information, but his sentences were always unfinished from failure of memory, and want of words to express himself; his general health was impaired, and his appetite poor. After having been for some time under medical treatment, it was determined to try if he could be induced to work at his business, another tailor being constantly employed in the same division with him. Some difficulty occurred in persuading him to make the effort; but by encouraging language, and holding out the hope to him that he should be enlarged as soon as he shewed that he was able to resume his trade, he was led to begin. At first his progress was slow, and frequently interrupted by fits of abstraction: but he gradually became more collected, and capable of longer continued application, and by steady perseverance in this course he recovered perfectly, and was discharged nine months after his admission.—A man, aged twenty-eight, who had served for some years in the East India Company's Artillery, was invalided in the island of St. Helena, and discharged as unfit for service in consequence of insanity, which had commenced with delirium tremens. He was admitted into the asylum about twelve months after being first attacked, when he was incapable of giving any correct account of himself; he was melancholic, and in general silent; when he did speak, his conversation was an incoherent jumble of the recollection of past events; his general health was a good deal impaired. For nearly three months after his admission there was very little change in his situation, he then began to shew more intelligence, and to take more notice of what was passing around him. It was discovered that he had been accustomed to the care of horses, and was fond of them; advantage was taken of this circumstance, and he was employed about the manager's horse, which in a short time was entrusted entirely to his care. From this period he steadily improved, and his recovery was complete in twelve months from the time of his admission. After being discharged as a patient, this man was employed as a keeper in the asylum; the duties of which situation he has now performed in a very satisfactory manner for the last two years. I may here mention, that one of the best nurses at present in the establishment is a woman who first entered it as a patient many years ago, and was then in a state of violent excitement.

The example of others employed around them greatly assists in overcoming the disinclination to every kind of exertion which many patients evince; and although the results of employment will not be always as satisfactory as in the examples I have adduced, still in every case more or less advantage will be derived from it. The general health is promoted, discipline is maintained, and active exertion in the open air is found more advantageous in producing quiet sleep than opiates. Many of our most industrious patients are persons whose cases are likely to prove incurable, but who are made as happy as their several states admit of; some, by their labours, are fully requiting, others materially lessening, the expense of their maintenance to the public."

### Twentieth Report of the Director of the West Riding of York Pauper Lunatic Asylum.

We have lately devoted some space to the consideration of insanity, and of the questions that hang by it. The utility of a periodical journal of medicine consists, we imagine, in bringing important subjects prominently, and in succession, before the public eye, and in directing particular attention to the main practical points connected with them. We would fain believe that by pursuing such a plan, we are contributing, and not meanly, to the diffusion of science, if not to the advancement of it.

The Report before us, brief at the best, is peculiarly so in reference to the medical history and management of insanity. But we are vaguely promised, at some future period, the information we may reasonably ask for.

The number of patients in the house appears to range between 317 and 366. The average number during the year was 346. Of these, rather more than one half were males. All cases of insanity, favourable or unfavourable, are admitted, a circumstance that must tell, of course, on the mortality.

*Statement of the Admissions, Discharges, and Deaths of all the Patients in the Asylum, since its Opening, on the 23rd of November, 1818.*

| | | | | |
|---|---|---|---|---|
| Patients admitted within three months after the first attack .. .. | 788 | Patients discharged cured, who had not been insane more than three months before admission | 424 | Dead 188 |
| Patients admitted within twelve months after the first attack .. .. | 457 | Patients discharged cured, who had not been insane more than twelve months before admission .. .. .. | 246 | Dead 168 |
| Patients admitted who had been insane from one to thirty years.. .. | 584 | Patients discharged cured, who had been insane from one to many years before admission, | 62 | Dead 296 |
| Patients admitted who have had previous attacks, and have been confined before in this Asylum .. .. | 346 | Patients discharged cured, who have had previous attacks.. . | 392 | Dead 156 |
| Patients admitted who are stated to have had previous attacks, but who have not been confined here .. .. | 405 | Patients not cured, discharged by desire of their friends, and others by order of the Magistrates .. .. .. .. | 282 | |
| | 2580 | | 1406 | 808 |

We think the following Table of Re-admissions both curious and likely to be profitable. Some perhaps may feel surprise at the preponderating number of re-admissions, after a considerable period.

*Statement of Patients re-admitted.*

| | MALES. | FEMALES. |
|---|---|---|
| Patients re-admitted who had not been discharged three months .. .. .. .. .. .. .. .. .. .. .. .. | 23 | 22 |
| Patients re-admitted who had been discharged between three and six months. .. .. .. .. .. .. .. .. .. | 23 | 17 |
| Patients re-admitted who had been discharged between six and nine months .. .. .. .. .. .. .. .. .. .. | 9 | 16 |
| Patients re-admitted who had been discharged between nine and twelve months .. .. .. .. .. .. .. .. .. | 15 | 13 |
| Patients re-admitted who had been discharged between one and two years .. .. .. .. .. .. .. .. .. .. | 34 | 27 |
| Patients re-admitted who had been discharged between two and three years .. .. .. .. .. .. .. .. .. .. | 17 | 25 |
| Patients re-admitted who had been discharged between three and ten years .. .. .. .. .. .. .. .. .. .. | 52 | 45 |
| Total.. | 173 | 165 |

Of 183 admitted in 1838, 45 are the subjects of insanity, the result of intemperance; 38 with propensity to suicide, having made actual attempts on their lives; 16 are epileptic, and 8 idiots from birth.

There are one or two points on which we shall touch.

1. *Bad Effects of great Reduction in Mania.* "It is satisfactory to find, that notwithstanding the great increase in the number of patients, (the average throughout the year being 346,) the mortality has diminished. Thirty-seven have died, some within a short period of admission. Amongst this number was a patient who had been insane seven days before being sent here; he had been bled from the arm three times, to syncope; the quantity abstracted was 96oz. in less than four days; from this, and 'severe purgation,' the system was so reduced, that the patient, as may be expected, never rallied. Another was received in a sinking state, with the hands and arms enormously swollen, of a blackish colour, approaching to mortification, and a deep indentation in the upper arm, produced by a cart-rope, which had been used for the purpose of binding him down in bed; he had been highly excited, but on his admission all restraint was removed; he became calm, answered every question rationally, and continued in that state up to the period of his death, which took place in twelve hours."

2. *Sketch of the Economy of the Asylum.* The patients are all paupers, their respective parishes paying for each 6s. per week. This sum defrays every expense. They are fed, lodged, and clad alike, wearing a dress of grey woollen cloth, which is woven and made up by themselves; they rise at six A. M. in the Summer, and seven in the Winter, and all who are in a fit state, (of whom there are a great number,) attend with such servants as can be spared at morning prayers precisely at eight o'clock. They breakfast on milk pottage and bread at half-past eight. At nine o'clock the gardener, farmer, laundry woman, &c., select those patients, who by previous arrangement with the Director have been fixed on, for their several occupations, and commence work.

At eleven, the workers have a luncheon of bread, and three-quarters of a pint of table-beer. They dine at one. Their dinners are one day, meat, yeast dumplings, and potatoes, and half-a-pint of beer; the next, soup, with potatoes and dumplings, alternately.

At two, work is resumed, and at four a luncheon is distributed, similar to that in the forenoon. At seven they have supper, of milk pottage and bread. At eight, the bed-room doors and window shutters are carefully locked, the clothes folded, and placed on the outside of each door. The resident physician, (who is director) the matron, the apothecary, and housekeeper, reside in the Institution. They not only visit each patient once a-day, but are constantly amongst them. The two visiting physicians attend twice a-week each, and more frequently if necessary. The two visiting surgeons once a-week, and as often as there are surgical cases requiring their attendance.

The number of patients employed will be seen from the following table:—

| | |
|---|---|
| Sewing and knitting .. .. .. .. .. | 61 |
| Household work .. .. .. .. .. .. .. | 78 |
| Agriculture .. .. .. .. .. .. .. .. | 45 |
| Shoemakers .. .. .. .. .. .. .. .. | 5 |
| Weavers .. .. .. .. .. .. .. .. .. | 6 |
| Baking and brewing .. .. .. .. .. .. | 4 |
| Joiners, painters, coopers, and blacksmiths, | 7 |
| Washing .. .. .. .. .. .. .. .. .. | 18 |
| Picking Coir .. .. .. .. .. .. .. .. | 74 |
| Total .. .. .. .. | 298 |

Here, as elsewhere, the utility of labour would appear to have been proved.

### MARYLEBONE INFIRMARY.

On Long-Continued Contraction of the Lower Extremities, from an Affection of the Spine. By R. A. Stafford, Esq. Surgeon to the St. Marylebone Infirmary, &c.

We shall allude to some cases detailed by Mr. Stafford, before we notice the views he has founded on them. The cases are five in number.

*Case* 1.—Jan. 1833.—Mary Kean, æt. 20, a slight-made girl, and of small stature, was admitted into St. Marylebone Infirmary with complete contraction of the right lower extremity, and contraction of the third and little finger of the hand of the same side. She possesses the full power of feeling in both limbs, but the knee-joint is firmly bent and stiff; the calf of the leg rests upon the back part of the thigh; and the heel presses closely upon the nates, the foot being turned backwards. She states that she has been the subject of epileptic fits ever since her infancy; that about two years ago she was seized with violent pain in the spine in the lumbar region; pain in the hip and knee; and extreme pain along the course of the ischiatic nerve, accompanied with considerable tenderness and increased sensibility of the whole limb. At this period the lower limb became gradually and firmly contracted, and no means employed could prevent it. Some little time afterwards the two last fingers also became contracted, and thrown across the two first on the hand of the same side. She was admitted into one of the London hospitals, where the symptoms became mitigated, but the limbs remained the same.

For the last two years, excepting the contraction, she has continued pretty well, but at present she is suffering from her original symptoms. The pain in the back, hip, and knee, is extremely acute; and the tenderness and sensibility of the whole limb is so great that she cries out at the slightest touch, and she cannot even bear the weight of the bed-clothes upon it. On tracing and pressing upon the ischiatic nerve, it is extremely painful and tender. Her pulse is quick, fever high, tongue furred, skin hot and dry, and thirst great. She has constant restlessness and sleepless nights.

These symptoms continued, being more or less violent, for three or four months; and the treatment adopted was, frequent abstraction of blood from the lumbar region by cupping; the application of leeches along the course of the ischiatic nerve; the exhibition of antimonials, opiates, and aperients, as often as required, and according to the necessity of each remedy. When the chronic stage commenced, counter-irritants were employed and frequently repeated, both on each side of the spine and along the course of the ischiatic nerve, in the form of blisters and the tartar-emetic ointment. Lastly, issues were made and kept open for a considerable time on each side of the spine in the lumbar region.

When the inflammatory symptoms had entirely subsided, the pain in the back had ceased, and the tenderness of the limb had diminished, an attempt was made to unbend the contraction, and bring the limb into its natural state. To effect this purpose small thin pillows were first introduced between the calf of the leg and back part of the thigh, drawing them as near as possible to the flexure of the knee-joint. At first this caused extreme pain, and occasioned several attacks of epilepsy. By degrees, however, the patient was able to bear the treatment. Thicker pillows were then employed, and, as before, occasioned great pain, but which, after the limb was accustomed to them, subsided. When the pillows had been worn for some time (six weeks or two months) the contraction has relaxed sufficiently for a machine to be introduced between the two limbs, and which is so contrived that, by turning a screw, the leg can be gradually extended. It consists of two splints joined by a hinge at the flexure of the knee-joint—one resting on the back part of the thigh, and the other on the calf of the leg; a bow made of iron, the extremities of which rest upon each splint, and through the centre of which passes a screw, which is also attached to the angle at the junction of the splints. A nut (as it is termed) is turned on the screw, and presses on the centre of the bow; consequently when the centre is pressed upon, the two extremities extend the splints.

With this machine the limb was gradually extended inch by inch, until at length the leg and thigh were as straight as natural. During the progress of the extension, friction was employed, and the knee was frequently steamed. The limb was then exercised; and after the period of seventeen months from the beginning of the treatment to the termination of it, the patient was discharged from the hospital cured. The fingers likewise were gradually extended, and returned to their natural use and position.

This case is one of some interest. We have given it in the words of the author, as it is not susceptible of useful abbreviation. The disease on which the contraction of the leg and of the fingers depended, was probably seated in the spine. The existence of epileptic fits from infancy furnishes collateral evidence of lesion of the nervous centres. The affection was probably inflammatory, from the severity and course of the pain, and from the *pyrexia* which attended it. The case is peculiarly instructive, because, to use the words of Mr. Stafford, it shews, that, however firm and complete a contraction of this description may be, yet by perseverance and care, and the gradual and equal employment of extension, the limb can be restored to its healthy action.

We pass over the second case, and pause at the third.

*Case* 3.—"A young lady, æt. 18, of a strumous habit, fell into bad health. She first found that she had partially lost the power over her legs; she then was seized with violent spasms and involuntary twitchings of the lower extremities.

At length these spasms increased, and became so confirmed that the legs were half drawn up in a semi-flexed position, and would remain so, unless they were put down again by another person. At this period of the complaint the case was considered as chorea, and treated as such. No improvement, however, took place; the spine, therefore, was examined, and it was found that five or six vertebræ were diseased, and that there was angular curvature in the dorsal region of the spine, beginning at the fourth dorsal vertebræ. The friends of the patient would not consent to any treatment, consequently the curvature increased, and the contraction of the legs likewise. She remained in this state for a few months, when she came under my care. I found her as above described, and my first object in the treatment was to get the limbs back to their natural position. Leeches were frequently applied to the back, and counter-irritants employed. Under this treatment she improved, some consciousness of feeling in the legs returned, and the contraction became sufficiently relaxed to enable me to place her on one of Mr. Earle's beds; but if the limbs were touched or moved they immediately retracted. After she had been under my care for a twelvemonth, improving in health, and there being some slight amendment both in the power of feeling and of motion in the legs, her friends became extremely impatient at the tediousness of the case; they made an attempt, without my consent, to get her up; employed friction, and exercised the limbs. The spasms returned in a violent degree; they extended on to the arms and all over the body. At length she became comatose, and in this melancholy state died. No examination of the body was made."

*Case* 4, that of a lad aged 15, is one of angular curvature of the spine, beginning at the fifth dorsal vertebra, and ending at the tenth. He states that about three years ago, when stooping down, a young man in play jumped upon his back and knocked him down. From this time he felt pain in the spine, and the curvature commenced. About two years ago his lower limbs became in one week contracted, and drawn up in a semi-flexed position, so that the heels are within four inches of the nates, and they have remained so ever since. They can be straightened to a certain extent, but immediately they retract as if by a spring with great force. The treatment has been abstraction of blood from the spine and counter-irritants, &c. At length the contraction subsided, the limbs could be straightened, and the boy is now (July 3d) walking about like other people.

*Case* 5.—In this, dissection was obtained, and the nature of the lesion ascertained with precision. Ann M'Cartney, æt. 41, died in St. Marylebone Infirmary, September 7th, 1838. Nearly three years previous to her death, her lower extremities became firmly contracted, so that the heels almost touched the nates. After this she became insane, and was an inmate in the Lunatic Asylum at Hoxton. On examination of her body the arachnoid membrane of the spinal marrow was thickly studded along its whole course by specks of bony matter, consisting of patches of various sizes. These bony concretions had spiculæ on their surfaces, which, from their roughness, must have been a constant source of irritation, if not inflammation of the spinal cord itself.

Having related these cases, we may now pass to the views engrafted on them. It will probably appear that those views outrun the data.

"In many cases," says Mr. Stafford, "which have been termed Local Hysteria—the symptoms of which Sir Benjamin Brodie has so ably described, in a work lately published by him—I have every reason to believe, if they were traced to their original source, that the medulla spinalis and its membranes would be found to be the seat of the disease. I am induced to come to this conclusion from the symptoms of these affections. For instance, one individual shall wholly or partially lose the sensation of a limb; another shall wholly or partially lose the power of volition; a third shall have a limb contract, as in the cases

I shall presently mention; and others, again, shall have extreme pain in the hip, the knee, or ankle, accompanied with extreme tenderness and sensibility, and even swelling, and yet have no actual disease of either joints. Some also shall have extreme tenderness of the spine itself; so much so, that they shall flinch even at the slightest touch. Retention of urine, tympanitis, and many other symptoms, shall affect others, and all which appear to me to be alone traceable to the nerves belonging to the parts themselves, or to the grand trunk from whence they spring.

When we reflect on these various affections—loss of sensation—loss of motion—increased sensibility—spasm and contraction of muscles—we are led to inquire how such different phenomena can be produced. It appears to me that the different functions which the brain and medulla have to perform, will explain the whole. First, the medulla spinalis governs the power of feeling in the trunk and limbs; therefore, if feeling be lost, it must necessarily arise from some affection of the cord: secondly, it governs motion; consequently if motion be lost, it has suffered some functional derangement, or morbid alteration of structure: thirdly, if contraction of the limbs takes place, we know that the medulla spinalis governs the action of muscles, and therefore that most probably the affected part is the spine: fourthly, if there be increased sensibility of a limb, as in the cases which I shall presently relate, there can be but little doubt that it is produced from some morbid condition or inflammation of the nerve itself, or of that part of the substance of the medulla which governs sensation, from whence it has its origin: and, fifthly, when contraction and spasm; inflammation of that part of the substance of the cord which governs motion, or at the origin of the nerve which supplies the muscles of the affected part. When we compare these affections with the injuries of the spine, we may observe a great similarity in the symptoms of both. When paraplegia is not complete in concussion of the spine, there may be partial loss of feeling or of motion in the lower limbs. The same occurs in what has been termed local hysteria. A sense of numbness, or partial paralysis, is often complained of by the patient, and I have known individuals thus affected drag one leg from this cause. The same feeling of disordered sensation I have observed takes place in the upper extremities, and which sometimes happens in injuries of the spine without the lower suffering. It is not uncommon, also, in injuries of the spine for the patients to complain, as they do in local hysteria, of severe pain in the hip, the knee, and the ankle. A case of this kind occurred some time ago in St. Bartholomew's Hospital. A man threw himself from a high wall, having the impression that it was falling. He injured his spine in the lumbar region, and partially lost the feeling of one leg, having also retention of urine, and inability of retaining the fæces. When he was admitted into the hospital he referred the chief of his sufferings to severe pain in the hip, the knee, and the heel, accompanied with extreme tenderness and sensibility of the whole limb. He was relieved by abstraction of blood from the lumbar region and moxas; but he returned again, after his discharge, three times, suffering from the same symptoms, before he ultimately recovered.

Contraction of the limbs is occasionally produced from an injury of the spine. An instance of this kind occurred a few years ago at Penkridge, in Staffordshire. A man fell from off a waggon-load of hay, and fractured the spine at the second, third, and fourth lumbar vertebræ, they being considerably displaced laterally. He was paralyzed below the injury for a considerable period, although now he is partially recovered; but the arms became contracted, and so firmly that the humeral part of them rests fixed to the side—the fore-arm on the humeral part, and the hand on the fore-arm, with the fingers firmly clenched. The cases which I shall presently relate very much resembled this, without an accident occurring.

Tympanitis frequently occurs in local hysteria. In concussion of the spine we see the same. Tympanitis is a frequent symptom attending it. Again total or partial retention of urine occurs in local hysteria. The same happens in injuries of the spine also; and if the bladder be sufficiently long paralyzed in the former affection, the same morbid changes of the organ occur; the mucous membrane is found inflamed, and the urine fœtid and decomposed."

After some remarks upon tympanitis, to which we need not refer more particularly, Mr. Stafford proceeds:—

"To continue, however, with the comparison of the similarity of local hysteria with affections of the spine, let us observe the symptoms where there is visible disease of the vertebral column. In these cases we see the same class of symptoms: total or partial paralysis of the lower extremities; loss of motion, or of feeling individually, or in an equal ratio; spasms and cramps in the muscles; paralysis of the bladder causing retention of urine; loss of power of the rectum; and inaction of the bowels. How commonly, also, do we find that the patients complain of pain in the hip, the knee, and the ankle, and refer their sufferings rather to one of these joints than to the spine, where there is evident disease!

Contraction of the lower limbs likewise not unfrequently occurs where there is angular curvature, examples of which I shall presently relate. In some cases, also, there may be extreme tenderness and increased sensibility of the legs, and more particularly in the feet. On the contrary, in others there may be a sense of numbness and dead weight, although the locomotive powers are complete.

In addition to these symptoms, how frequently do we find, when destruction of the vertebræ has taken place, and the curvature is advancing, that the patients, and particularly females, suffer from symptoms resembling inflammation of the viscera! Hence they may have acute pain and extreme tenderness in the region of the liver, the stomach, and intestines; and bearing all the characters of hepatitis, enteritis, gastritis, and peritonitis. When we consider the intimate and frequent connexion of the medulla spinalis with the sympathetic, and the various organs this latter nerve supplies, it is not difficult to account for any symptoms which may occur; it is natural to suppose, if any portion of the cord be affected by disease, from pressure, irritation, or deviation from its normal course, that not only the nerves immediately derived from it, but those collaterally connected with it, would suffer derangement of function. Such, then, being the similarity of the symptoms of these affections, with those where there is real and perceptible injury and disease of the spine, it appears to me fair to infer that they arise from some functional derangement or morbid condition of the medulla spinalis."

We notice these views not simply on their own account, but because they are ramifications of the doctrine of spinal irritation, which has become rather fashionable.

Mr. Stafford, it will be observed, uses a certain chain of argument to shew that "local hysteria"* is identical with spinal affection—and he supports that argument by cases. Those cases, at least some of them, are certainly instances of spinal affection, attended with pains and contraction of the muscles of the extremities. But we do not hesitate to affirm that those cases are not fair instances of "local hysterical affection," and therefore prove nothing with regard to *its* nature. On this point then we join issue with our author. His cases, we repeat, may be instances of spinal affection, but they are *not* instances of hysteria.

We therefore turn to his argument. The gist of it is, that the various symp-

---

* There cannot be a doubt of the impropriety of the application of the term "hysteria" to the local nervous affections in question. But the term *is* applied, and provided that all understand its force and meaning, it matters little what the term may be.

toms of hysteria find their analogue (we use the phraseology which is now the mode) in the symptoms of spinal disease. Picking out particular symptoms, as Mr. Stafford does, such an analogy may be perceived. Nor can we wonder that it is so. The spinal marrow is a portion of the great nervous centre. Its affections, and other affections of the nervous system, will naturally, then, display the features of family resemblance.

But while we grant that there may be analogy between the symptoms of the one disorder and the other, it does not follow that there may not be differences as striking as the similarities, and such differences there are.

If, in spinal disease, there is loss of motion, or of feeling, that loss is of a more or less permanent character. It is *not* absent one minute and present the the next. It is *not* present if the patient's attention is directed to it, and gone if she is diverted from thinking of it. It is *not* present before marriage, and magically dispersed by a husband or accouchment. It is *not* unaccompanied by pyrexia or by symptoms of grave implication of some important organ. It is *not* attended with symptoms of the most heterogeneous character, aping all diseases, permanently retaining the impress of none. It is *not* seen in the multitudes of young girls, whom it leaves after a while in comparative health, to play their parts as wives and mothers.

All these points of dissimilarity, points which mark the grand distinction between a malady which has and which has not a habitat in some vital organ, Mr. Stafford has omitted to notice. Were such a line of argument permitted, were such reasoning received and deemed conclusive, we might, in turn, pronounce hysteria, general or local, to be each of the diseases that it simulates. It would be organic uterine disease—"white swelling"—diseased spine—insanity —hepatitis—diseased lungs—the whole nosology.

We feel assured that this chimera of "spinal irritation," or spinal alterations, as the essence of hysterical affections, will, after its due season, pass away, and be buried in the crowded grave of pathological fancies.

---

ROYAL INFIRMARY OF EDINBURGH CLINICAL REPORT. BY J. SYME, Esq.*

We extract a few cases from this interesting Report.

1. *Dislocation of the Thigh-bone on the Dorsum Ilii, of nine weeks' standing, reduced.* W. S. aged 36, a mason in Dunfermline, fell while walking on the road, from his foot becoming fixed in a cart track, and dislocated his left thigh-bone upwards. He applied to a bone-setter in the neighbourhood, and also to a regular practitioner, who attempted without success to effect reduction. He then proceeded to a famous bone-doctor near Perth, who told him that the injury, having existed so long, nearly five weeks, could not be remedied. He was now recommended to Mr. Syme.

The patient possessed a strong muscular frame, and the bone had become very moveable in its new position. So far the circumstances were unfavourable, and rendered the prospect of success still less promising than it appeared to be, from nearly nine weeks having elapsed since the accident happened. It being still considered right to make an attempt, the day after his admission, on the 6th of December, the patient, after losing sixteen ounces of blood from the arm, was put into the warm bath for an hour. He was then carried into the theatre and took at intervals a solution containing four grains of tartrate of antimony. He lay upon his right side, with a mattress between him and the floor. A hair

---

* Edinburgh Med. and Surg. Journ. Oct. 1838.

cushion was placed in the perineum, over which and obliquely round the pelvis a broad canvas band was passed, and fastened to a ring in the wall. A skein of worsted being then secured to the thigh immediately above the knee by the *clove hitch*, extension was effected by the aid of pullies nearly in the direction which the limb had acquired through the displacement of the bone. At the end of 40 minutes reduction was effected without any snap or perceptible grating. He recovered quickly, and shortly after his dismissal, walked fifteen miles without difficulty.

2. *Dislocation of the Thigh-bone upon the Dorsum Ilii, of six weeks' standing—reduction.*—E. W. aged 26, admitted on the 17th of July, having met with the dislocation six weeks previously.

"She was admitted at 11 A. M. and immediately placed in the warm bath, where I found her on making my visit at 12. As her muscular system did not seem strong, or likely to afford much resistance, I proceeded to attempt reduction without any further preparation, except administering a dose of tartrate of antimony. The process was conducted as has been described above, and at the end of ten minutes proved successful, the bone returning into its place with a dull grating sensation."

Another case is detailed. In all of them, the extension was not maintained continuously, but completely relaxed from time to time, in order to fatigue the muscles, and disturb the patient's involuntary efforts to resist the exertions for his relief.

Two cases of dislocated shoulder-joint occurred in the course of the Winter, respectively of two and three weeks duration. They were both easily remedied by extending from the wrist in the direction of the long axis of the trunk, so as to prevent the *latissimus dorsi* and *pectoralis major* from opposing the resistance which they are apt to do when extension is made transversely. We think this latter observation practically very valuable.

3. *Fortunate Treatment of Fracture at a late period.*

In his observations on a case of fracture of the thigh-bone which was made to unite, after having been treated unsuccessfully for six months, Mr. Syme observes:—

"About two years ago, there was a case of this kind in Perth, which attracted considerable attention. The patient, a gentleman between thirty and forty years of age, was treated by a surgeon of great experience and respectability, for a fracture of the thigh, during six weeks. At the end of this time, the limb was taken off the double-inclined plane on which it had been laid, and found to be shortened as well as distorted, very much in the same degree and direction as in the case just related. The patient then proposed a consultation, which was opposed by his attendant, on the ground that no good could be done by any mode of treatment at that distance of time from the injury. He therefore placed himself under the care of another practitioner, who sent for me to consider with him what should be done. I suggested the same plan that has been described above, and was happy to hear that, through the assiduous care of Dr. Malcolm, it soon had the effect of rendering the injured limb perfectly straight and strong."

The plan to which Mr. Syme alludes is neither more nor less than the careful application of the long splint.

4. *Why torn Arteries do not bleed.*

"The amputated limb afforded a good example of the mode which I have repeatedly described in former reports as the true explanation of torn arteries not bleeding. The internal and middle coats of the vessel were not irregularly lacerated or disposed so as to obstruct the passage through it, but presented a smooth circular edge, round and beyond which the external or cellular coat had collapsed in the form of a conical bag filled with coagulum."

5. *Fistula in Ano—Operation unsuccessful from not includiug the Internal Aperture in the Incision.*

Donald Gunn, aged 31, from Cape Wrath in Sutherlandshire, came to Edinburgh last January, on account of a *fistula in ano*, from which he had been suffering two years and a half. He was admitted into a public institution, and subjected to an operation, eight days after which he was dismissed as cured.* Finding that his cure consisted in his being worse than ever, he applied at the Edinburgh Infirmary.

On examination it appeared that a deep gash had been made into the rectum. The edges of the wound were swelled, and shewed no disposition to heal. Searching for the cause of this, Mr. S. found an internal aperture at some distance to one side of the incision, which, probably, from being made towards the summit of the sinus, was much deeper than necessary, and in a wrong direction. He divided the parts that lay between the internal aperture and the wound, after which the patient quickly got well.

"For a number of years past I have endeavoured to direct attention to the important observations of M. Ribes in regard to the general existence and uniform position of the internal opening of *fistula in ano*. From the case just related, and many others that might be mentioned, it seems that sufficient care is still not bestowed on this point of practice. In the course of last winter I saw a man of rank and consequence who had been operated upon seven years before by a surgeon of this city for *fistula in ano*. He suffered deep incisions and a two months' confinement, but did not get quit of his complaint. Sinus after sinus formed, and he despaired of ever being well. I found an internal opening, made the necessary incision, and sent the patient home perfectly sound at the end of a fortnight."

Mr. Syme must be owned to have been very lucky. It has not been our fortune to see patients perfectly sound in a fortnight however perfectly the sinus was divided. No doubt there is often much unnecessary gashing, and often as much bungling. It is to draw attention to the fact that the internal aperture of fistula is almost always low in the gut, that we have noticed this case.

6. *Extraordinary Operations for Hæmorrhoids in Edinburgh.*

"About three months ago, a gentleman, between twenty and thirty years of age sent for me, in the hope that I might be able to suggest something for his relief, and thus related the sad history of his sufferings.

In July 1837, he visited a watering-place about thirty miles from Edinburgh. While there he one day had a long walk after taking medicine, and, in consequence, perceived a fold of skin at the orifice of the anus swelled and painful. This external pile, being a new ailment to him, so excited his apprehension as to induce an immediate return to town. He travelled in a carriage, and of course aggravated the complaint by doing so. A surgeon was sent for, who applied leeches and poultices for *four* days, and then intimated the necessity of an operation, which he accordingly performed. This was scooping out a portion of the gut, an inch and a-half in length.

The cavity was stuffed with a sponge, and the patient's legs were tied together. He was not able to turn himself in bed for three weeks, being all this time he said stupified with pain and the large opiates that were administered to him. His urine was all taken away by the catheter. At the end of two months he was

* How any man could be pronounced cured in a *fortnight* after the operation for fistula passes our comprehension.—*Rev.*

able to go down stairs, and then got his dismissal to the country, with instructions to prevent the anus from contracting too much by *occasionally introducing his finger into it.* But notwithstang all the care that could be used by a most respectable practitioner, who, in compliance with instructions sent to him, attempted to introduce tallow candles, and had some made on purpose of a most diminutive size, the orifice became smaller and smaller, until it would hardly admit a quill.

The patient returned to town, and he supposes had incisions made to enlarge the opening, at least he felt acute pain, and saw blood flow. Weiss's instrument for dilating the female urethra was then introduced, and screwed open for half an hour with excessive agony. This operation was repeated every second day for four months. He then went to the country, and passed a bougie every third or fourth day. Finding no improvement at the end of three months he had returned, in despair of ever regaining his health. He had no evacuation of his bowels unless by taking medicine; and when the desire to discharge the thin matters contained in the gut came upon him he had no power to restrain their escape. He felt constant uneasiness and burning pain about the anus, and through the whole pelvis. He was thin and haggard-looking, and unable for any exertion of body or mind."

Comment on such a case is superfluous. The singular thing is, that according to Mr. Syme :—

"The fact is, that removal of the extremity of the rectum has of late years been taught and practised in this city, as the best mode of treating those hemorrhoidal affections which are generally comprehended under the title of *prolapsus ani.*"

---

### Dundee Lunatic Asylum.

### Eighteenth Report of the Directors.

The pages of our present Number will afford evidence not only of the general interest felt on the subject of insanity, but, also of our own desire to promote the general amount of knowledge on its characters and treatment.

The leading objects of the Report before us would appear to be;—first, the confirmation of the value of employment of the insane—secondly, the recommendation of public worship. The Report is not long, at least the medical part of it is not, and we shall present what portions seem to be likely to prove useful.

1. The number of admissions during the past year has exceeded that of former years, has outrun, indeed, the accommodation.

Of those admitted several were insane before but had been cured—such cases are always more difficult to treat than others. The powers of the brain seem, by repeated attacks, to become gradually weaker, leaving the individual at last in a silly, childish, idiotic state. At least one half of those relapses have supervened without any known cause, and nearly the whole of the remainder can be distinctly traced to intemperance in the use of ardent spirits.

A young girl, upwards of fifteen years of age, was admitted, labouring under hysterical mania of three or four years' duration. The cause of the disease in this instance, though rather uncertain, is supposed to be a fright. Her symptoms are remarkable. While sitting in the room, and without the slightest perceptible cause, she will spring up and roar in the most terrific manner, and as suddenly become quiet, and either sit like a statue, apparently unconscious, or speak calmly and intelligently. When she makes this noise she hideously distorts her features. She is very often vicious, and so powerfully violent that she has been known to clear the apartment of all the other patients.

A young man who was obliged, from his violence, to be restrained for some time, requested, after he became convalescent, to be allowed to sleep with one part of the confinement near him in bed, and much to his gratification this was agreed to. On being questioned, he gave for a reason, that when the article was near him he felt confident of being perfectly secure.

2. *Remittent Mania.*—There are in the Asylum several old cases of remittent mania. In many of these cases the actions of the system are exalted to an extraordinary degree, and continued for a long period before nature becomes exhausted. The paroxysms generally come on without any very evident cause. As they are, however, more frequent in some cases in Summer than Winter, it is probable that heat has some effect in producing them. They vary too in their kind and duration. During the paroxysm the strength, even of old persons, is astonishingly great,—wakefulness, loquacity, obscenity, violence, fætor, &c., with a peculiarly vivid expression of eyes and countenance, are the most prominent symptoms. These paroxysms sometimes continue, without intermission, for days, weeks, or months, baffling all attempts to allay the excitement. But some of this unfortunate class of patients seem happy amidst their turmoil, and mischief—they appear absolutely overjoyed, singing and talking in the merriest strain possible, and in many respects resemble the drunkard when in a certain stage of excitement. By-and-bye a change suddenly takes place in their appearance—the pulse falls, the eye loses its lustre, they become quieter, heavy, sleepy, exhausted, stupid, and partially unconscious; they will then dose for days, perhaps weeks, passing their motions, involuntarily, requiring wine, and the most nourishing food, with the utmost attention, to keep them alive. Soon after gaining a little strength, the same state of excitement is suddenly reproduced, to be succeeded by the like depression, and this course will go on for years, till death puts an end to the scene. Nothing can be more remarkable or striking than the changes which take place in these lunatics. A stranger seeing them in their different states would scarcely believe that they were the same persons.

Patients whose malady intermits become fat and stout in the absence of their paroxysms; other lunatics, who are only occasionally irascible and obstreperous, are quickly relieved by medicine, baths, and seclusion, and generally in a day or two are fit to resume their work. On the appearance of premonitory symptoms they are immediately removed from where they could do mischief to themselves or others.

3. *Causes.*—"We have great difficulty, and do not always succeed, in procuring a proper history of every case. The cause of the malady, in particular, is very frequently concealed from us; but, if we deduct the nine from the moral causes who are also hereditarily affected, we find, in those admitted, that the physical causes are the most productive of insanity. But in both classes the result is, that nearly one half have had an hereditary tendency to this disease. The slightest cause, or rather what would not at all affect others, is quite sufficient to produce the malady in those who derive the disorder from their parents. In some families the tendency is so strong that we have several times had more than one member of them."

4. *Treatment.*—"As a general rule every individual case requires a different plan of treatment. Various remedies are employed; but we find, as stated in former reports, that there is no specific for the cure of insanity. Topical blood-letting is of the greatest service, so is dry cupping. Blisters, and a liniment composed of the tincture of cantharides, the spirit of hartshorn, and croton oil, applied twice or thrice to the shaven scalp have also proved beneficial. General blood-letting is very rarely resorted to. Baths of all kinds and cold lotions are in constant requisition, and are used with great advantage. Calomel, jalap, salts, rhubarb, tartar-emetic, colocynth, and croton and castor oils are in general use. The grand principle of moral treatment consists in directing the mind of the patient from the subject or subjects on which it is deranged to those on which it is not. As reason is disordered, it is obvious that all attempts to restore it by merely discussing with the insane the subjects on which it is disordered must fail. No appeal to fact *versus* fiction proves useful; a self-evident proposition frequently, or rather generally, appears to them to be absurd. It requires, therefore, much firmness, much caution, and a clear judgment, as well as quick perception, in conjunction with the best feelings of our nature, to bring moral treatment to bear successfully on the mind of the lunatic. Occupation offers many advantages; and idleness to the insane, as well as to those of sound mind, is itself a punishment; and to that portion of the monomaniacs who converse rationally on ordinary subjects, and generally conduct themselves with propriety, occupation is both an amusement and a powerful agent in recovery. Perhaps the good effects of this plan are best seen in the violent lunatic immediately after he has been subjected to the necessary medical treatment. To the ordinary maniac it has been of great service; to those bordering on fatuity it has also done good; and we may indeed add, in all cases where tried it has proved in some degree beneficial. The very bustle, excitement, and change, that our manufacturing and other works create in the House do good; which is also in some measure felt by those lunatics whose high rank and education prevent them from joining in these healthful exercises. It must, however, be admitted that medical as well as moral treatment succeeds in a much greater degree in the recent than in the old cases. No accident whatever has occurred, since the introduction of this plan of moral treatment in 1830, notwithstanding all kinds of tools and implements are daily used by the patients. In addition to the healthful exercises in which the ladies and gentlemen engage, such as music, dancing, and walking into the town and neighbourhood, and—which have been alluded to before—they are permitted to go into the country for several hours, in open or close carriages, according as the weather, &c. permits. In regard to restraint, moral treatment may itself be considered as a means of restraint, for it has generally superseded the other articles used for that purpose. To apply restraint properly is a most difficult and most painful, though in some cases an absolutely necessary task; and no one can execute this delicate business well who has not been accustomed to the treatment of the insane. It requires great humanity, great experience, and decided skill in the application of it. We have seen it have the most extraordinary good effect, owing in a great measure to the way it was applied."

The following is an interesting case.

In the Spring of last year a cabinetmaker was admitted labouring under deep-rooted melancholy, who had been nearly twelve months affected. We tried all kinds of treatment, and at last by our perseverance have been entirely successful. In September he asked for his tools, they were immediately procured—a bench was got—and wood purchased; he shed tears—always said he was just going to begin to work, but something mental always prevented him, and he continued in this state till last April, when his depression almost left him, and he began to work in earnest. Since that time he has made handsome chests of drawers, &c., and has been cured, not more to the delight of his relatives than of us. This case is instructive, and shows how much can be done by a persevering plan of treatment, even with patients whose malady is of long duration. Another lunatic who was bred a flesher, after he became convalescent, made himself generally useful, and slaughtered and cut up the pigs for the use of the Institution.

5. *Public Worship.*—" In this country the first institution which gave to its inmates the benefit of religious instruction was the Glasgow Royal Lunatic Asylum, and this was done, on the recommendation of its physician, nineteen years ago. In 1831, divine service was introduced here, the members of the Presbytery taking the duty Sabbath about, until a regular chaplain was appointed. Every succeeding Report has truly stated that nothing but good has resulted from its introduction, and that it has been and is highly appreciated by the lunatics in this Asylum, and to these statements we gladly bear testimony; and it is gratifying to learn that the other private and public asylums which have followed the good example set by the Glasgow one, confirm what is here stated—thus showing not only the great utility, but the safety, of public as well as private worship, to those labouring under lunacy."

Such are the main features of the Report, which we may observe emanates from Dr. Nimmo and Dr. Mackintosh. We trust that they will endeavour to render the Institution as beneficial to the profession and to science as possible. Careful reports are of immense service.

---

### EXPERIENCE OF MR. PORTER IN PUNCTURING THE DURA MATER AFTER TREPHINING.

The following remarks are extracted from a Lecture on the Trepan, by Professor Porter, in our clever contemporary, the Dublin Medical Press.

Suppose, he says, that the trepan has been applied, and neither blood nor matter found—shall the dura mater be opened to search for the cause of mischief? Setting aside reasoning and other authority, Mr. Porter states, and we quote the facts with which he is actually acquainted.

" I must say that both my reason and experience are directly opposed to the operation. I have seen it performed, and performed it myself. In one instance of compression, the trepan was applied over the seat of the injury, or rather on the spot that had received the blow. Immediately on the piece of bone being taken out, the dura mater and brain rose into the vacant space, as if suddenly relieved from some compressing force, and (what seemed to decide the operator on puncturing the dura mater,) the brain did not exhibit the usual phenomenon of pulsation. The membrane was cautiously divided, but nothing found; and dissection shewed afterwards, that the extravasated blood was situated at the base of the brain. In the month of December, 1836, I trepanned a man for symptoms of compression, the result of inflammation, and apparently produced by matter. On removing the bone, I found that a scale had been broken from the internal table, and wounded the dura mater, and on this being taken away a small quantity of pus pumped up through the little aperture. I thought that if there ever was a case to justify proceeding farther, it was this one; the membrane had already been wounded, and I had ocular demonstration of the existence of matter underneath. I, accordingly, enlarged the opening, but I took nothing for my pains, no more matter was evacuated, and it was found, after death, that it could not have been, for it was smeared over the surface of the dura mater, closely adherent to it, and by no possibility capable of being removed. In a former lecture I stated that there was no circumstance to justify a surgeon in cutting through the dura mater, and I am strongly disposed to repeat the precept here."*

---

* Dublin Medical Press, Feb. 13th, 1839.

### Clinical Observations on the State of the Heart and on the Use of Wine in Typhous Fever. By William Stokes, M.D. &c.*

The object of Dr. Stokes is to determine with precision the symptoms or circumstances which indicate the exhibition of wine in typhous fever. We may observe, in limine, that Dr. Stokes repudiates the doctrine of exclusive solidism, the modern theory that fever is but the symptom or effect of some local lesion.

"There can be no doubt," he says, "that the typhus of Great Britain and Ireland is a disease of the whole system, not symptomatic of any particular local lesion; shewing on the one hand a tendency to a favourable termination, after a period which varies indefinitely; and on the other, being capable of destroying life *with* various lesions, or *without* any appreciable change in the solids. It is a disease on which anatomy sheds but a negative light, not telling us what it is but rather what it is not.

With respect to the organic lesions, I consider them as much secondary to the general disease, as the pustule in small-pox is to the disease of variola. Their not unfrequent absence in the worst cases of the disease proves that they are not the cause of typhus, while in cases where they do occur, we observe a signal want of proportion between their amount, and the severity of the symptoms. They are in the fullest sense inconstant in their seat and extent, incompetent to the explanation of symptoms, and unnecessary to the characteristic phenomena of the disease."

After some remarks on the greater frequency of intestinal ulceration in the fevers of the Continent and those of Great Britain and Ireland, Dr. Stokes proceeds to observe:—

"If we compare the inexperienced man with him who has had a long continued practice in fever, we may often observe that the former employs a too vigorous antiphlogistic treatment in the commencement of the disease, and delays the exhibition of stimulants until the powers of life are sunk too low, while the latter is much more cautious in husbanding the strength of his patient, and shews much less fear of resorting to wine and other stimulants. It is in determining on the use of wine in fever that the junior or inexperienced man feels the greatest difficulty; it is in its exhibition that he betrays the greatest uncertainty and fear. This is to be explained by referring to the general character of the doctrines which have prevailed within the last quarter of a century, and which are only now beginning to yield to a more rational pathology. The doctrine of an exclusive or almost exclusive solidism, which referred all diseases to visible changes of organs, which taught that inflammation was the first and principal morbid phenomenon, and that fevers were always the result of—or accompanied with—some local inflammation, was, however disguised under various denominations, the doctrine taught to the majority of our students. Their ideas were thus exclusively anatomical; inflammation formed the basis of their limited pathology, and thus instructed, they entered on the wide field of practice, most of them having never even attended a fever hospital; utterly ignorant of the nature of essential fevers, they applied, in the diseases of debility, the treatment of acute local inflammation, and delayed stimulation until nature could not be stimulated."

But the question comes—"what distinctive symptoms call for wine—how shall the inexperienced practitioner know when it is required?" Dr. Stokes thinks that to the want of wine the loss of many lives may be ascribed, and the very object of his paper is to endeavour to furnish some signs by which we may learn to give or to withhold stimulants.

* Dublin Journal, March, 1839.

It has long been known that if, under the use of wine, the pulse diminishes in frequency, the prognosis is favourable—if, under the same circumstances it becomes more rapid, our anticipations grow more gloomy. Reflection on this point led Dr. Stokes to endeavour to ascertain, whether any appreciable condition of the heart, besides the frequency of its contractions, would furnish the indication that was wanted. This inquiry he pursues with great zeal and with much interest through his lengthened paper. We cannot follow him throughout his observations and his cases, but we must content ourselves with stating the results, and recommending our readers most earnestly to consult the original paper. The conclusions of Dr. Stokes are:—

1. That the condition of the heart in typhous fever must be determined by the application of the hand and stethoscope, the pulse being an uncertain guide.

2. That a diminished impulse, or a complete absence of impulse occurs in certain cases of typhous fever.

3. That in such cases we may observe a diminished first sound, or even an absence of the first sound.

4. That both these characters may exist with a distinct pulse.

5. That although in most cases the diminution of the impulse and first sound coexists, yet that impulse may exist without corresponding first sound, and conversely, that the first sound may be heard although unaccompanied by impulse.

6. That these phenomena are most evident as connected with the left side of the heart.

7. That when the impulse and first sound are lessened or lost, the return to healthy character is observed first over the right cavities.

8. That in some cases both sounds are equally diminished.

9. That in few cases the first sound preponderates.

10. That these phenomena indicate a debilitated state of the heart.

11. That they may occur at an early period of the disease, and thus enable us accordingly to anticipate the symptoms of general debility.

12. That the existence of these phenomena, in a case of maculated adynamic fever, may be considered as pointing out a softened state of the heart.

13. That this softening of the heart seems to be one of the secondary local lesions of typhus.

14. That the diminution or cessation of impulse, the proportionate diminution of both sounds, or the preponderance of the second sound, are direct and nearly certain indications for the use of wine in fever.

